# Heavy Metals in Infant Clothing: Assessing Dermal Exposure Risks and Pathways for Sustainable Textile Policies

**DOI:** 10.3390/toxics13080622

**Published:** 2025-07-25

**Authors:** Mei Xiong, Daolei Cui, Yiping Cheng, Ziya Ma, Chengxin Liu, Chang’an Yan, Lizhen Li, Ping Xiang

**Affiliations:** 1 Yunnan Key Laboratory of Plateau Wetland Conservation, Restoration and Ecological Services, National Plateau Wetlands Research Center, Southwest Forestry University, Kunming 650224, China; 18334273684@163.com (M.X.); daolei_cui@126.com (D.C.); yipingcheng2001@126.com (Y.C.); maziya2023@126.com (Z.M.); liuchengxin2024@163.com (C.L.); 2 Yunnan Research Academy of Eco-Environmental Sciences, Kunming 650034, China; ycaandy88@163.com; 3 Planning and Research Center, Kunming Academy of Ecological and Environmental Sciences, Kunming 650032, China; ynllz@126.com; 4 Yunnan Provincial Key Laboratory of Public Health and Biosafety, Kunming 650500, China

**Keywords:** heavy metals, health risks, human skin keratinocytes, oxidative stress, textile safety

## Abstract

Infant clothing represents a critical yet overlooked exposure pathway for heavy metals, with significant implications for child health and sustainable consumption. This study investigates cadmium (Cd) and chromium (Cr) contamination in 33 textile samples, integrating in vitro bioaccessibility assays, cytotoxicity analysis, and risk assessment models to evaluate dermal exposure risks. Results reveal that 80% of samples exceeded OEKO-TEX Class I limits for As (mean 1.01 mg/kg), Cd (max 0.25 mg/kg), and Cr (max 4.32 mg/kg), with infant clothing showing unacceptable hazard indices (HI = 1.13) due to Cd (HQ = 1.12). Artificial sweat extraction demonstrated high bioaccessibility for Cr (37.8%) and Ni (28.5%), while keratinocyte exposure triggered oxidative stress (131% ROS increase) and dose-dependent cytotoxicity (22–59% viability reduction). Dark-colored synthetic fabrics exhibited elevated metal loads, linking industrial dye practices to health hazards. These findings underscore systemic gaps in textile safety regulations, particularly for low- and middle-income countries reliant on cost-effective apparel. We propose three policy levers: (1) tightening infant textile standards for Cd/Cr, (2) incentivizing non-toxic dye technologies, and (3) harmonizing global labeling requirements. By bridging toxicological evidence with circular economy principles, this work advances strategies to mitigate heavy metal exposure while supporting Sustainable Development Goals (SDGs) 3 (health), 12 (responsible consumption), and 12.4 (chemical safety).

## 1. Introduction

**Figure 1:**
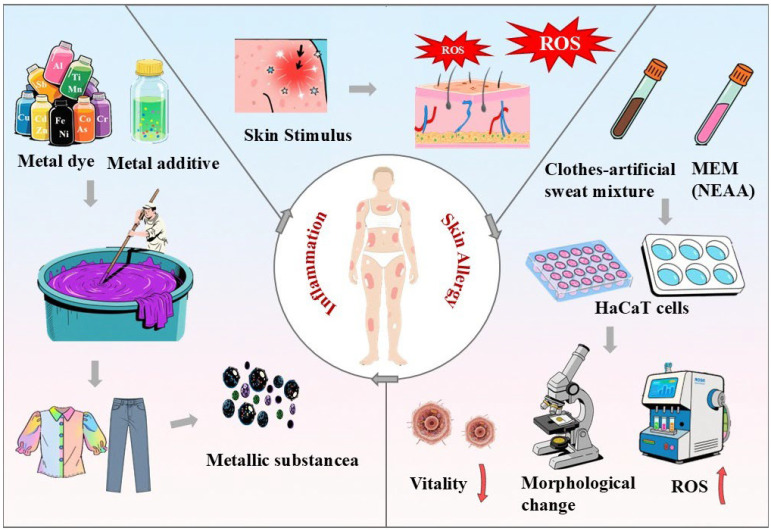
The influence of heavy metals in clothes on human health. Heavy metals in clothes lead to the generation of reactive oxygen species (ROS).

The global textile industry directly exposes consumers to chemical contaminants through prolonged skin contact [1,2], yet heavy metal transfer via clothing remains poorly quantified. Heavy metals including arsenic (As), lead (Pb), cadmium (Cd), chromium (Cr), cobalt (Co), copper (Cu), mercury (Hg), titanium (Ti), nickel (Ni), zinc (Zn), and ferrum (Fe) are inadvertently introduced into clothing during manufacturing processes, primarily through functional additives and processing auxiliaries. Sb serves as a flame-retardant catalyst in polyester, Cr and Cu act as mordants and metal-complex dyes (especially in black polyamide and colored cotton), while Ti and Zn are used in moisture-wicking treatments [3,4]. Synthetic fabrics exhibit distinct contamination patterns—black polyester consistently accumulates Cr and Ti, whereas dark-colored textiles (black/gray/navy) show higher Cr/Co levels compared to light colors (pink/red) [5,6,7]. These metals originate from dyes (e.g., Fe in black, Cu in blue, Al in pink) and finishing agents, creating heterogeneous exposure sources [8,9]. Of particular concern is the presence of heavy metals in clothing, which poses potential hazards to human health [5,10] (Figure 1).

Clothing-borne heavy metal may transfer via perspiration-mediated dissolution or physical abrasion processes, creating potential dermal contact risks during extended wear periods [4,11,12]. Although only 5–30% of clothing-associated metals migrate into sweat, chronic exposure through dermal absorption poses significant risks [3,6]. Prolonged skin contact facilitates bioaccumulation of biologically available forms, particularly for sensitizing metals (Cr, Co, Ni, Cu) linked to dermatological reactions [13,14]. Notably, Ti (abundant in synthetic fabrics) demonstrates a hazard quotient (HQ) > 1 via dermal exposure, while Sb in polyester reaches an HQ of 0.3, indicating potential systemic effects [5,15]. Current safety standards (e.g., OEKO-TEX^®^) fail to address real-world exposure scenarios. Rovira et al. [7] documented metal concentrations in retail garments exceeding ecological thresholds yet complying with textile regulations, highlighting a critical gap between permissible limits and actual health risks. This discrepancy underscores the need for exposure-based risk assessment, particularly for vividly colored and synthetic textiles where additive-derived metals concentrate.

Due to infants’ physiological characteristics including thinner epidermal barriers, higher body surface-area-to-mass ratios, and frequent hand-to-mouth activity, even trace concentrations of heavy metals may pose disproportionate health risks. As these toxicants can permeate through dermal contact with clothing, they are particularly concerning for children given their heightened susceptibility to As and Cr exposure [16,17]. Similarly, only a limited number of studies have employed standardized procedures using a unified analytical method to simultaneously determine heavy metal concentrations across different material types (e.g., leather samples underwent microwave digestion in nitric acid and hydrogen peroxide before MP-AES detection [18] and ICP-OES analysis of textile-derived metals was performed post-nitric acid digestion [19]). These approaches would enable comparative analysis of the results. Nevertheless, clothes become saturated with sweat during prolonged and high-intensity physical activity, where extended skin contact coincides with elevated temperatures. These may accelerate heavy metal leaching from fabrics and subsequent dermal absorption, potentially elevating exposure risks. To better simulate such exposure conditions, the use of artificial sweat extraction for soluble heavy metals is methodologically justified [20,21,22]. Current research on toxic substances in apparel products remains critically limited. Consequently, this study was conducted to address critical safety concerns for both consumers and the textile industry itself.

This study systematically investigates heavy metal contamination in textiles by the following: (1) employing ICP-MS to quantify total and bioaccessible metal concentrations across 33 garment samples, (2) applying the European Chemicals Agency Guidelines on information requirements and chemical safety as well as the United States Environmental Protection Agency to evaluate infant-specific exposure risks, (3) elucidating cytotoxic mechanisms through oxidative stress analysis in HaCaT keratinocytes.

## 2. Materials and Methods

### 2.1. Collection and Preparation of Samples

In terms of clothing sample collection, we randomly purchased cost-effective clothing from different online market platforms. We then recorded the specifications of each sample, including color, material composition, and clothing type, and then systematically classified and labeled it. The brand-new unwashed clothes samples were subjected to a 48 h drying process in an oven maintained at 60 °C, after which each sample was precisely cut into 1 × 1 cm square slices using scissors and weighed to determine fabric density (Table 1).

The International Organization for Standardization (ISO 3071:2005) standard method was employed for pH assessment [23]. Clothing specimens (1.0 g) were cut into 2 cm × 2 cm pieces (*n* = 3) and immersed in 50 mL of pure water (27 °C) in 100 mL bottles. Following thorough wetting, the solutions were equilibrated with continuous shaking for 2 h before instrumental analysis.

### 2.2. Determination of Heavy Metals in Clothing

After oven-drying 0.5 g cloth specimens, microwave digestion was conducted with 10 mL concentrated HNO_3_ (65% Suprapur grade, E. Merck, Darmstadt, Germany) using a Milestone Start D Microwave Digestion System. The thermal protocol involved the following steps: (1) a 5 min temperature increase to 105 °C, (2) 15 min maintenance at 180 °C, and (3) 20 min terminal digestion at 200 °C. Cooled digests were membrane-filtered (0.45 µm) and volumetrically adjusted to 25 mL with deionized water before cryogenic storage (−20 °C). Elemental quantification was performed via inductively coupled plasma mass spectrometry (ICP-MS), with method validation incorporating reagent blanks and certified reference materials (spinach leaves, National Institute of Standards and Technology) for quality assurance.

### 2.3. Exposure Levels Through Direct Skin-Contact Clothes

Elemental concentrations detected in clothes were utilized for human exposure and risk evaluation, following the European Chemicals Agency Guidelines for Chemical Safety Assessment (ECHA) [24]. The assessment framework incorporated the following:

Exp_derm_ = C_cloth_ × 10^−6^ × d_cloth_ × A_skin_ × F_mig_ × F_contact_ × F_pen_ × T_contact_ × n/BW

C_cloth_ is the concentration of metal in clothing (mg/kg), 10^−6^ is a conversion factor, d_cloth_ is the cloth surface density (mg/cm^2^), A_skin_ is the area of contact between the cloth and the skin (cm^2^), F_mig_ is the fraction of metal migrating to the skin per day (1/d), F_contact_ is the fraction of contact area for skin (unitless), F_pen_ is the penetration rate (unitless), T_contact_ is the contact duration between cloth and skin (d), and n is the mean number of events per day (1/d). BW is the body weight. This study not only incorporates exposure dose parameters from the US Environmental Protection Agency (US EPA) but also considers this environmental context in the domestic apparel market (Table 2).

### 2.4. Framework for Evaluating Health Risks

The non-carcinogenic risk evaluation was conducted using the Hazard Quotient (HQ) approach, which evaluates potential health effects by comparing actual exposure levels with established reference doses. This model quantifies risks associated with chemical exposure through clothes contact, calculated as follows:

HQ = Exp_dermal_/RfD_dermal_
CR = Exp_dermal_× SF_dermal_
HI = ∑HQ

RfD_derml_ represents the reference dose for the dermal exposure pathway of non-carcinogenic heavy metals, while SF_dermal_ is the slope factor for the dermal exposure pathway of carcinogenic heavy metals (Table 3) [28]. According to the national standard, when HQ < 1, non-carcinogenic risk indices were maintained within the safe range and indicating negligible health concerns; when HQ ≥ 1, heavy metals pose a non-carcinogenic risk. CR is the carcinogenic risk, where CR < 1 × 10^−6^ represents no cancer risk; 1 × 10^−4^ < CR < 1 × 10^−6^ represents an acceptable cancer risk; CR > 1 × 10^−4^ represents an unacceptable cancer risk.

### 2.5. Artificial Sweat Extraction from Clothes and Cell Experiment

The artificial sweat was synthesized according to ISO 3160/2 specifications [29]. A standardized artificial sweat solution was prepared by the preparation of fresh artificial sweat, which involves dissolving lactic acid (0.1 wt%), urea (0.1 wt%), and sodium chloride (0.5 wt%) in 1 L of deionized water (Table 4). For the artificial sweat simulation experiment, 1 g of the pre-treated textile samples was weighed and placed in a 50 mL centrifuge tube. Then, 20 mL of freshly prepared artificial sweat solution was added. The mixture was incubated at 36 °C (simulating skin surface temperature), with shaking at 100 rpm for 24 h. Post-extraction, samples were filtered through 0.45 µm membranes after centrifuged (500 rpm, 10 min). The resulting supernatants were preserved at −20 °C before ICP-MS analysis of bioaccessible heavy metals (Cd, Cr, Cu, Mn, Ni, Zn, As). Blank samples, control samples, and certified reference materials are used to verify the accuracy of the instrumental method.

### 2.6. Cell Culture and Viability Detection

Human skin keratinocytes (HaCaT) were maintained in MEM (with NEAA) Basal Medium containing 10% fetal bovine serum and 1% penicillin–streptomycin under standard culture conditions (37 °C, 5% CO_2_). Cells were seeded in multiwell plates (6-well/96-well format) and cultured for 24 h before extract exposure.

HaCaT cells (a spontaneously immortalized human keratinocyte line) were plated in 96-well plates (1 × 104 cells/100 µL/well) and allowed to adhere for 24 h. Cells were exposed for 24 h to the clothing–artificial sweat extract, which was rendered suitable for cell culture applications by 0.22 µm filtration. Cell viability was quantified using CCK-8 reagent (10 µL/well, 2 h incubation at 37 °C), with measurements of optical density (OD) at 450 nm (Molecular Devices LLC, San Jose, CA, USA). Cellular morphology was documented using an inverted microscope (TS-100, Nikon, Tokyo, Japan). Cell viability (%) = [(OD exposed group − Mean OD blank group)/(OD control group − Mean OD blank group)] × 100%.

### 2.7. ROS Detection Method

HaCaT cells cultured in 6-well plates were treated with a control medium or various dust extracts for 24 h. Intracellular ROS levels were quantified using 2′,7′-dichlorofluorescein diacetate (DCFH-DA) as a fluorescent probe. After staining, cellular fluorescence intensity was measured for 10,000 events using flow cytometry (CyFlow^®^ Cube 6, Sysmex Partec, Nuremberg, Germany) with excitation/emission wavelengths of 488/525 nm. The generation intensity of ROS was expressed as a percentage of the control group. The source of the reagent kit is from Yfxbio Biotech Co., Ltd. (Nanjing, China).

### 2.8. Quality Control and Statistical Analysis

To ensure that no other factors affect the results and to minimize experimental errors, quality control measures must be implemented during the operation of this experiment. All procedures were conducted in accordance with standard QA/QC protocols. The determination of heavy metals in clothing samples included blank controls, clothing samples, and certified reference materials (GB/T 17593, China) [30]. The spike recovery rates for all target heavy metals fell within the acceptable range. All statistical analyses were performed using GraphPad Prism Version 7.0 software (GraphPad Software LLC, San Diego, CA, USA). Statistical significance was established at *p* < 0.05. The ICP-MS detection thresholds were as follows: 0.006 µg/kg for As, 0.003 µg/kg for Cd, Zn, and Cu, 0.002 µg/kg for Mn, 0.005 µg/kg for Ni, and 0.02 µg/kg for Cr.

## 3. Results and Discussion

### 3.1. Heavy Metal Level in Clothing Samples

Clothing-associated heavy metals present significant exposure risks through dermal absorption and accidental ingestion, with particular concern for vulnerable populations. Infants and individuals with cutaneous hypersensitivity represent particularly susceptible populations due to developmental vulnerabilities and compromised epidermal barrier function, respectively [5,31]. This research evaluated the concentrations of nine heavy metals (As, Cd, Cr, Cu, Mn, Ni, Pb, Zn, Fe) in clothing samples (Table 5) and compared them to the limit values of OEKO-TEX Standard 100 (Table 6) [32]. Analytical results revealed Cu (mean: 11.30 mg/kg), Zn (mean: 13.83 mg/kg), and Fe (mean: 31.68 mg/kg) as the most abundant metals, consistent with their use in textile dyes and pigments [33]. While these metals showed elevated concentrations, other toxic metals—particularly As (mean 1.01 mg/kg), Cd (max 0.25 mg/kg), and Cr (max 4.32 mg/kg)—are of greater concern due to their well-documented toxicity even at trace levels [34].

Notably, five toxic metals (As, Cd, Cr, Pb, and Ni) were found to exceed the stringent Class I limits (baby wear category) in clothes samples, highlighting significant exposure risks for infants. While most samples complied with Class II (direct skin contact) and Class III (occasional skin contact) standards for adult clothing, Cd and As exceedances suggest potential safety concerns for adults that warrant further investigation. These findings align with recent epidemiological evidence linking heavy metal exposure to developmental disorders in children [35], while generally supporting the adequacy of current standards for adult textile safety.

### 3.2. Hazard Quotient (HQ) and Carcinogenic Risk (CR) Assessment

The Hazard Quotient (HQ) serves as a critical indicator for evaluating non-carcinogenic health risks associated with dermal exposure to textile-borne heavy metals. This investigation systematically compares HQ values across three distinct garment categories (adult male, adult female, and infant clothing) to quantify differential exposure risks and identify vulnerable populations. Table 7 is HQs derived from the ratio of the level of skin exposure to RfD_dermal_ through skin contact of the garment with the detected element concentration for each textile sample. Most elements exhibited HQ values below the safety threshold (HQ < 1) across all garment types (except Cd), indicating acceptable risk levels for adult populations. Female garments (blouses and underwear) showed marginally higher HQ values compared to male counterparts (T-shirts and underwear), likely attributable to increased fabric–skin contact area, tighter fit enhancing dermal absorption, potential differences in fabric composition, etc. Baby clothing presented the highest hazard indices (HI = 1.13), with Cd being particularly concerning (HQ = 1.12). This exceedance of safety thresholds (HQ ≥ 1) suggests the following: heightened vulnerability due to infants’ greater surface area-to-body mass ratio, and potential for cumulative exposure through multiple contact routes.

Table 8 shows the carcinogenic risk assessment of clothing exposed to skin. While all CR values fall within the acceptable range (10^−4^–10^−6^). Two key findings warrant attention: (1) Cr emerges as the predominant carcinogenic concern (CR = 4.35 × 10^−5^ in infant clothing) and exhibits an approximately 27-fold-higher risk than As; (2) women’s shirts had a slightly higher risk than men’s T-shirts, with a difference of about 13.7%. The elevated risk observed in infant clothing aligns with previous studies demonstrating children’s heightened susceptibility to heavy metal exposure [36], while the gender differentials in adult clothing warrant further exploration of textile design factors influencing dermal contact.

### 3.3. Bioaccessibility of Heavy Metal in Artificial Sweat

As the primary protective barrier of the human body, skin serves as a crucial defense against environmental pollutants [37]. Clothing in direct contact with the skin often becomes moist with sweat, which may facilitate the leaching of metals from textiles and subsequent dermal absorption [38]. To accurately simulate dermal exposure scenarios, we employed acidic artificial sweat to assess the bioaccessible fraction of heavy metals in clothing textiles (Table 9)—a methodology aligned with ISO/EN standards for material safety testing. Our findings corroborate growing evidence that soluble metal species, rather than total content, drive metal-induced skin toxicity [39].

Quantitative analysis revealed significant leaching of heavy metals under simulated sweat conditions, with release levels following the order: Zn > Cu > Cr > Mn > Ni > As > Cd. Notably, bioaccessibility exhibited a distinct pattern of Cr > Ni > Mn >Zn > Cu > As > Cd, while Pb and Fe remained below detection limits. The high bioaccessibility of Cr (37.8%) is of particular concern, as it suggests disproportionate mobilization from textiles despite moderate total content. This observation is consistent with the research results that link soluble chromium to contact dermatitis and barrier dysfunction [11], strengthening its importance as a driver of the risk of clothing-related exposure.

### 3.4. Clothing Bioaccessible Extract Changed Cellular Morphology and Viability

**Figure 2:**
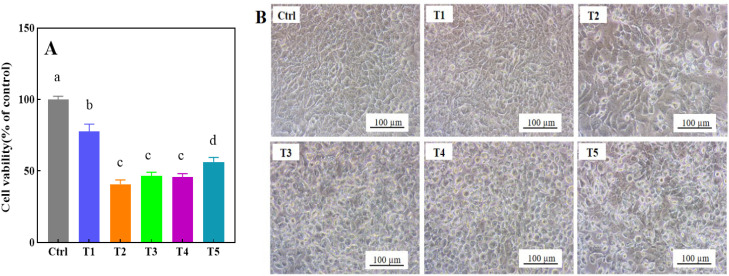
HaCaT cell morphology was examined following 24 h exposure to clothing extracts, with an inverted phase-contrast microscope at 200× magnification. Results are presented as mean ± standard deviation from three independent experiments. Columns marked with different letters indicate statistically significant differences (*p* < 0.05) compared to the control. (**A**) shows cell vitality, while (**B**) represents the cell morphology. T1–T5 indicate the sample numbers. Different letters (a, b, c, d) indicate significant differences between groups, while the same letters indicate no significant differences.

Keratinocytes, as the predominant epidermal barrier cells, represent an optimal model for evaluating dermal toxicity [40,41]. To investigate the potential health risks posed by bioaccessible metals released from clothing materials into artificial sweat, we systematically analyzed HaCaT cell viability and morphological alterations following exposure (Figure 2). Cell viability assays, a well-established metric for heavy metal toxicity assessment, revealed a significant time-dependent reduction in survival rates after 24 h treatment with the clothing extract (Figure 2A). Notably, the T2 group exhibited the most pronounced cytotoxic effect, with cell survival markedly lower than all other test conditions.

Cellular morphology, a sensitive indicator of physiological integrity and cytotoxic damage [42], displayed progressive deterioration across treatment groups. HaCaT cells exposed to higher concentrations (T3 and T4 groups) exhibited characteristic hallmarks of cytotoxicity, including loss of epithelial morphology, membrane blebbing, and increased cell detachment (Figure 2B). These morphological aberrations correlated strongly with the viability data, reinforcing the dose-dependent cytotoxic effects of the extract.

### 3.5. Clothing Extracts Triggered Cellular Oxidative Damage

**Figure 3:**
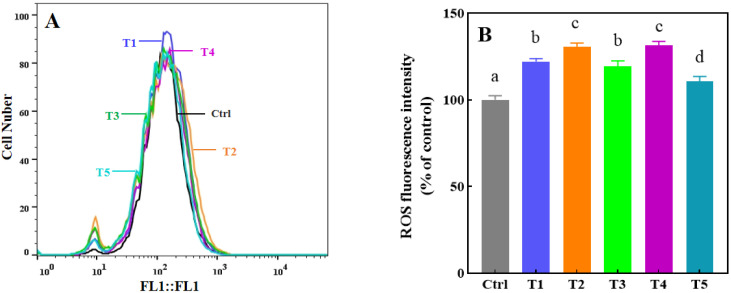
Oxidative stress analysis of HaCaT cells after 24 h exposure to clothing extract. Intracellular ROS levels were quantified through flow cytometric analysis and were expressed as the average fluorescence intensity (**A**), with results normalized to control group values (set as 100%) (**B**). Results are presented as mean ± standard deviation from three independent experiments. Columns marked with different letters indicate statistically significant differences (*p* < 0.05) compared to the control. T1-T5 indicate the sample numbers. Different letters (a, b, c, d) indicate significant differences between groups, while the same letters indicate no significant differences.

Oxidative stress represents a key molecular pathway through which heavy metals manifest their genotoxicity [43], and the intracellular concentration of reactive oxygen species [25] serves as a quantitative indicator of it severity [44]. A disruption in the equilibrium between ROS production and endogenous antioxidant defenses may result in pathological ROS accumulation, which can lead to cell dysfunction and ultimately may lead to cell oxidative death [45]. Consequently, we employed flow cytometric analysis to quantify ROS-associated fluorescence intensity in HaCaT cells following clothes extract exposure (Figure 3A,B). The results showed that HaCaT cells exposed to the T2 and T4 exposed groups increased fluorescence intensity by 130% and 131% compared to the control group, the T1, T3, and T5 exposed groups was a slight increase (117 ± 4.9%), which was similar to the change in cell viability, demonstrating evidence that oxidative stress-induced damage may be one of the factors leading to the decrease in cell viability.

This study demonstrates that exposure to the clothing extract significantly elevates intracellular ROS levels in HaCaT cells, which we hypothesize may be attributed to the high Cr content in the extract. This finding aligns with the established literature indicating that Cr triggers the mitochondrial apoptotic pathway via ROS overproduction, ultimately resulting in HaCaT cell apoptosis and skin barrier dysfunction [41]. Notably, beyond apoptosis, emerging evidence suggests that ROS may further contribute to Cr-induced cutaneous toxicity by activating the NLRP3 inflammasome or disrupting autophagic flux [46]. While the current work primarily focuses on Cr-mediated cellular damage, future studies employing multi-omics approaches (e.g., transcriptomics/proteomics) could provide deeper mechanistic insights by systematically mapping ROS-dependent inflammatory and autophagy networks. Such research will provide a more comprehensive understanding of the pathogenic mechanism of chromium in skin toxicity.

## 4. Conclusions and Suggestions

**Figure 4:**
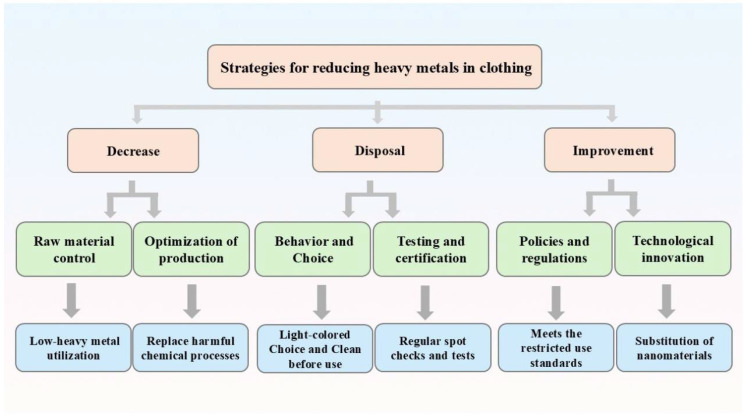
Strategies for reducing heavy metals in clothes. Strategies include the use of low-heavy metal raw materials, the improvement of laws and regulations, technological innovation, etc.

This study demonstrates that infant clothing serves as a significant dermal exposure pathway for cadmium (Cd) and chromium (Cr), with Cd exceeding hazard thresholds (HQ > 1) and Cr approaching carcinogenic risk limits (CR ≈ 10^−4^). The high bioaccessibility of Cr (37.8%) in artificial sweat and its role in oxidative stress-mediated cytotoxicity (131% ROS increase) highlight urgent gaps in textile safety regulations, particularly for synthetic, dark-colored fabrics. These risks disproportionately affect vulnerable populations in low-resource settings, where cost-driven manufacturing and lax enforcement converge. Towards sustainable textile systems to align with Sustainable Development Goal (SDG) 3 (health), SDG 12 (responsible consumption), and the Minamata Convention on Heavy Metals, we propose the following (Figure 4):

(1) Regulatory Upgrades: Revise OEKO-TEX Class I limits for Cd in infant wear and mandate bioaccessibility testing for Cr, especially in synthetic dyes.
(2) Green Chemistry Incentives: Subsidize non-metal dye alternatives (e.g., plant-based pigments) and adopt extended producer responsibility (EPR) frameworks.
(3) Global Harmonization: Strengthen labeling transparency across supply chains, prioritizing the EU’s REACH and ASEAN’s chemical safety protocols as benchmarks.
(4) Preventive measures: Wash new clothing thoroughly before use to eliminate heavy metal residues from dyes and avoid extended sweat saturation.
(5) Source control: Opt for natural fibers (cotton, hemp, wool) instead of high-risk synthetics like PVC-coated fabrics and avoid using electroplated accessories containing Cr or Cd.

## 5. Limitations and Future Directions

While this study focused on dermal exposure, future work should assess inhalation risks from textile microfibers and evaluate socioeconomic barriers to safer alternatives in low- and middle-income countries (LMICs). Community-engaged research is needed to tailor solutions to local production contexts. By integrating toxicological evidence with circular economic principles, this work advances a dual agenda: mitigating immediate health risks while fostering sustainable transitions in the global textile industry.

## Figures and Tables

**Table 1:** Basic characteristics and PH of clothing.

*n* = 33	Materials	Color	Density	pH
1	60% Cot; 33% PET; 7% PA	Pale yellow	16	7.90
2	100% PA	White	18	7.80
3	100% Ct	Orange	22	7.60
4	70% E; 30% PET	Black	12	8.00
5	90% Cot; 10% E	Grass green	28	6.48
6	100% Ct	Mint green	8	6.01
7	60% Vs; 21% PET; 19% PA	Blue	7	9.77
8	100% PET	Red	15	5.67
9	80% Vs; 20% PA	Dark gray	32	3.48
10	100% PA	Brown	15	7.71
11	74% E; 26% PA	Pink	14	6.57
12	100% Cot	Pink and white intersect	11	6.88
13	62% PET; 38% PA	Light orange	7	7.73
14	55% Vs; 45% E	Purple	6	6.99
15	90% PET; 5% PA; 5% E	Pale yellow	17	6.89
16	100% Ct	White	13	7.00
17	57% Vs; 23% E; 20% PA	Pale yellow	14	3.95
18	100% Cot	Indigo	26	7.67
19	40% E; 30% PET; 30% PA	Black	18	7.09
20	65% Cot; 25% PET; 10%Vs	Baby blue	36	6.15
21	100% Vs	White	5	6.55
22	66% PA; 44% PET	Sky blue	12	6.82
23	100% PET	Dark red	16	7.13
24	70% Cot; 15% PA; 15% PET	Khaki	21	6.99
25	100% Ct	Dark orange	23	6.48
26	73% E; 27% PA	Lilac	25	6.54
27	100% PA	Modena	26	7.44
28	91% E; 9% PET	Navy blue	16	8.13
29	100% Cot	Charcoal	19	8.28
30	100% PET	Maroon	13	7.30
31	60% PET; 20% E; 20% PA	Rose	14	7.28
32	100% Ct	Denim blue	22	7.56
33	70% Cot; 25% PA; 5% E	White	33	7.34

**Table 2:** Human exposure and risk assessment parameters.

Variable	Implication	Value	Unit	Data Source
d_cloth_	Cloth surface density	Table 1	mg/cm^2^	Present study
C_cloth_	Concentration of element in cloth	Table 5	mg/kg	Present study
F_mig_	Fraction of metal migrating to the skin per day	0.005	1/d	[4]
F_pen_	Fraction of penetration inside the body	0.01 (As 0.03)	Unitless	[25]
F_contact_	Fraction of contact area for skin	1	Unitless	[3,7]
T_contact_	Contact duration between skin textile	1	d	Assumed
BW	Body weight of an adult male Body weight of an adult woman Infants under 1 year old	70 60 6.98	70 60 6.98	[26,27]
A_skin_	Skin area of an adult male (T-shirt/underwear) Skin area of an adult woman (blouse/underwear) Skin area of infants under 1 year old (One-piece pajamas)	7120/1980 6941/1723 2754	cm^2^	[27]
n	Mean number of events per day	1	d	Assumed

**Table 3:** RfD_dermal_ and SF_dermal_ used in human health risk assessment.

Element	RfD_dermal_ (mg·kg^−1^·d^−1^)	SF_dermal_ (kg·d·mg^−1^)
As	3.00 × 10^−4^	1.50 × 10^0^
Cd	5.00 × 10^−8^	-
Cr	7.50 × 10^−5^	2.00 × 10^−1^
Cu	4.00 × 10^−2^	-
Mn	1.40 × 10^−1^	-
Ni	8.00 × 10^−4^	-
Fe	7.00 × 10^−1^	-
Zn	3.00 × 10^−1^	-

Notes: - Indicates no parameter.

**Table 4:** Formula for artificial sweat.

Reagent	Lactic Acid	Urea	Sodium Chloride	Deionized Water.
Content	0.1 wt%	0.1 wt%	0.5 wt%	1 L

**Table 5:** The total concentrations of heavy metals in clothing (mg/kg).

	As	Cd	Cr	Cu	Mn	Ni	Pb	Zn	Fe
Mean	1.01	0.16	1.98	11.30	2.16	1.51	0.19	13.83	31.68
S.D	0.30	0.04	1.12	5.14	0.60	0.71	0.09	6.96	17.79
Maximum	1.87	0.25	4.32	23.92	3.61	3.45	0.47	27.38	83.85
Minimum	0.53	0.09	0.23	2.80	1.24	0.62	0.07	3.18	4.84

Notes: S.D: Standard deviation.

**Table 6:** Oeko-Tex Standard 100 limit values (mg/kg).

Heavy Matals	As	Cd	Cr	Cu	Mn	Ni	Pb	Zn	Fe
Baby wear (I)	0.2	0.1	1.0	25.0	-	1.0	0.2	-	-
With skin contact (II)	1.0	0.1	2.0	50.0	-	4.0	1.0	-	-
Without skin contact (III)	1.0	0.1	2.0	50.0	-	4.0	1.0	-	-
Accessories	1.0	0.1	2.0	50.0	-	4.0	1.0	-	-

Notes: - Indicates no parameter.

**Table 7:** Non-carcinogenic risk of clothes.

Element	HQ				
	Male T-Shirt	Male Underwear	Woman Blouse	Woman Underwear	Infants
As	9.22 × 10^−4^	2.56 × 10^−4^	1.05 × 10^−3^	2.60 × 10^−4^	3.58 × 10^−3^
Cd	2.88 × 10^−1^	8.00 × 10^−2^	3.27 × 10^−1^	8.12 × 10^−2^	1.12 × 10^0^
Cr	2.49 × 10^−3^	6.94 × 10^−4^	2.84 × 10^−3^	7.04 × 10^−4^	9.68 × 10^−3^
Cu	2.67 × 10^−5^	7.42 × 10^−6^	3.04 × 10^−5^	7.53 × 10^−6^	1.04 × 10^−4^
Mn	1.42 × 10^−6^	3.96 × 10^−7^	1.62 × 10^−6^	4.02 × 10^−7^	5.53 × 10^−6^
Ni	1.75 × 10^−4^	4.86 × 10^−5^	1.99 × 10^−4^	4.94 × 10^−5^	6.78 × 10^−4^
Pb	2.41 × 10^−6^	6.69 × 10^−7^	2.74 × 10^−6^	6.79 × 10^−7^	9.33 × 10^−6^
Zn	4.05 × 10^−6^	1.13 × 10^−6^	4.61 × 10^−6^	1.14 × 10^−6^	1.57 × 10^−5^
Fe	4.05 × 10^−6^	1.12 × 10^−6^	4.60 × 10^−6^	1.14 × 10^−6^	8.27 × 10^−7^
HI	2.91 × 10^−1^	8.10 × 10^−2^	3.31 × 10^−1^	8.22 × 10^−2^	1.13 × 10^0^

**Table 8:** Carcinogenic risk of cloth.

Element	CR				
	Male T-Shirt	Male Underwear	Woman Blouse	Woman Underwear	Infants
As	4.15 × 10^−7^	1.15 × 10^−7^	4.72 × 10^−7^	1.17 × 10^−7^	1.61 × 10^−6^
Cr	1.12 × 10^−5^	3.12 × 10^−6^	1.28 × 10^−5^	3.17 × 10^−6^	4.35 × 10^−5^

**Table 9:** Bioaccessibility of heavy metals in clothing in artificial sweat (mg/kg).

	As	Cd	Cr	Cu	Mn	Ni	Pb	Zn	Fe
Mean	0.13	0.01	0.75	1.67	0.60	0.43	-	2.77	-
S.D	0.17	0.01	0.55	0.73	0.54	0.18	-	1.79	-
Maximum	0.16	0.02	1.33	3.18	1.68	0.64	-	5.03	-
Minimum	0.07	0.01	0.06	0.63	0.06	0.19	-	0.33	-
Bioaccessibility	12.91%	6.33%	37.78%	14.78%	27.63%	28.51%	-	20.02%	-

Notes: S.D: Standard deviation.

## Data Availability

All data analyzed or generated are included in this article.
